# Flipping the Script About Aging Through Films: The Gerontological Movie Database Review

**DOI:** 10.1093/geroni/igab046.386

**Published:** 2021-12-17

**Authors:** Sara Hackett

**Affiliations:** University of South Florida , Wesley Chapel, Florida, United States

## Abstract

Education about the heterogeneity of the older adult population is an important step for reducing ageist attitudes. As many undergraduate students view gerontology as an unrelatable discipline, educators are tasked with identifying innovative strategies to make course content engaging. The purpose of this presentation is to share an emerging educator’s experience with creating a novel essay assignment. Based off the International Movie Database (IMDb), the Gerontological Movie Database (GMDb) Review encourages students to use their knowledge to evaluate how older adults are portrayed in films. Explicitly, students must 1) choose a film that focuses on older adult characters and 2) apply key gerontological concepts (e.g., the life course perspective) to critique the film’s representation of aging. Though movie reviews are not a typical genre of writing, this assignment increases students’ understanding of how their perception of aging, coupled with master narratives embedded within today’s culture, influences the construction of age.

